# A new species of Rain Frog (Brevicipitidae, *Breviceps*) endemic to Angola

**DOI:** 10.3897/zookeys.979.56863

**Published:** 2020-10-27

**Authors:** Stuart V. Nielsen, Werner Conradie, Luis M. P. Ceríaco, Aaron M. Bauer, Matthew P. Heinicke, Edward L. Stanley, David C. Blackburn

**Affiliations:** 1 University of Michigan-Dearborn, Dearborn, Michigan, USA University of Michigan Dearborn United States of America; 2 Florida Museum of Natural History, University of Florida, Gainesville, Florida, USA University of Florida Gainesville United States of America; 3 Marquette University, Milwaukee, Wisconsin, USA Marquette University Milwaukee United States of America; 4 Port Elizabeth Museum (Bayworld), Humewood, Republic of South Africa Port Elizabeth Museum Port Elizabeth South Africa; 5 Nelson Mandela University, George, Republic of South Africa Nelson Mandela University George South Africa; 6 National Geographic Okavango Wilderness Project, Wild Bird Trust, Republic of South Africa National Geographic Okavango Wilderness Project NA South Africa; 7 Museu de História Natural e da Ciência da Universidade do Porto, Porto, Portugal Museu de História Natural e da Ciência da Universidade do Porto Porto Portugal; 8 Museu Nacional de História Natural e da Ciência, Lisboa, Portugal Museu Nacional de História Natural e da Ciência Lisboa Portugal; 9 Villanova University, Villanova, Pennsylvania, USA Villanova University Villanova United States of America

**Keywords:** Afrobatrachia, Anura, *Breviceps ombelanonga* sp. nov., cryptic species, multilocus, novel species, Sub-Saharan Africa, África Subsahariana, Afrobatrachia, Anura, *Breviceps ombelanonga* sp. nov., espécies crípticas, espécies novas, multilocus

## Abstract

Recent molecular phylogenetic work has found that *Breviceps* Merrem, 1820 comprises two major clades, one of which, the *B. mossambicus* group, is widely distributed across southern sub-Saharan Africa. This group is notable for harboring abundant cryptic diversity. Of the four most recently described *Breviceps* species, three are members of this group, and at least five additional lineages await formal description. Although *Breviceps* has long been known to occur in Angola, no contemporary material has been collected until recently. The three most widespread taxa, *B. adspersus*, *B. mossambicus*, and *B. poweri*, may all occur in Angola, but accurate species assignment remains challenging given the rampant morphological similarity between these taxa, and, until recently, the lack of genetic resources. Phylogenetic, morphological, and acoustic analyses of recently collected samples from disparate localities within Angola provide evidence for an undescribed species that is sister to *B. poweri*. The new species can be diagnosed from its sister taxon by lacking pale spots along the flanks, a pale patch above the vent, and a short, dark band below the nares (all present in *B. poweri*). Additionally, the male advertisement call differs from the three other *Breviceps* that might occur in Angola in having both a longer interval between consecutive calls and a higher average dominant frequency. We here describe this lineage as a distinct species, currently only known from Angola, and discuss the presence of other *Breviceps* taxa within Angola.

## Abstract (pt)

Investigações moleculares recentes revelaram que o género *Breviceps* Merrem, 1820, é composto por duas linhagens principais, uma das quais, o grupo *B. mossambicus*, é amplamente distribuído na região sul da África subsaariana. Este grupo é notável por albergar uma abundante diversidade críptica. Das quatro espécies de *Breviceps* recentemente descritas, três pertencem a este grupo, e pelo menos outras cinco linhagens adicionais aguardam a sua descrição formal. Apesar de o género ser conhecido de Angola desde há muito tempo, só muito recentemente foram colhidos novos espécimes. Os três taxa mais amplamente distribuídos, *B. adspersus*, *B. mossambicus* e *B. poweri* podem todos, porventura, ocorrer em Angola, no entanto a correta identificação destas espécies têm sido problemática devido às semelhanças morfológicas extremas entre este taxa, e, até muito recente, a completa ausência de material genético. Análises filogenéticas, morfológicas e acústicas dos espécimes recentemente colhidos em diferentes locais de Angola apontam para a existência de uma espécie nova para a ciência, irmã de *B. poweri*. A nova espécie pode ser diferenciada do seu táxon irmão pela falta de marcas pálidas nos flancos, mancha pálida acima do ventre e pequena banda negra abaixo do nariz (presentes em *B. poweri*). Para além destas características, o chamamento dos machos difere das outras três espécies de *Breviceps* que podem ocorrer em Angola por ter um maior intervalo entre chamamentos consecutivos e uma maior frequência média dominante. Descrevemos aqui esta linhagem como uma espécie distinta, atualmente apenas conhecida de Angola, e discutimos a presença de outras espécies de *Breviceps* em Angola.

## Introduction

*Breviceps* Merrem, 1820 is a genus of fossorial frogs widely distributed across southern sub-Saharan Africa, from Angola in the northwest, through Zambia, the southern portions of the Democratic Republic of the Congo and Tanzania, and southward throughout virtually all of southern Africa (Minter 2004; Minter et al. 2017). It currently comprises 18 species, although a recent molecular phylogenetic study indicates that this is an underestimate (Nielsen et al. 2018). Six species have been described since 2003 (Minter 2003; Channing and Minter 2004; Channing 2012; Minter et al. 2017), largely representing cryptic taxa embedded within what were previously considered widespread species or species complexes, namely *B. mossambicus* Peters, 1854 and *B. adspersus* Peters, 1882 (Nielsen et al. 2018). The justification for recent descriptions has largely been variation in nuptial call characteristics, geography, and mitochondrial genetic distances, yet many additional distinct genetic lineages have been identified and await formal description. Large-scale taxonomic revision is required but this remains problematic due in large part to limited genetic sampling (Nielsen et al. 2018), especially in the northwestern extent of the genus in Angola.

The taxonomy of Angolan *Breviceps* has long been problematic. Bocage (1870, 1873) was the first to report *Breviceps* in Angola based on two specimens from “Biballa” (currently Bibala, Namibe Province) that he referred to *Breviceps gibbosus* (Linnaeus, 1758). After receiving more specimens from other localities in Huambo and Huíla provinces, Bocage (1895) provided a more detailed description of the Angolan material and assigned all of these records to *B. mossambicus*. He noted that compared with other *Breviceps* (which, at the time, included only three species), Angolan specimens lacked a heavily granular dorsum (vs. granular in *B. verrucosus*) and had a continuous dark gular patch (vs. paired patches in *B. adspersus*). Unfortunately, the majority of these specimens were lost in the 1978 fire that destroyed the Lisbon Museum (Almaça 2000; Marques et al. 2018). Subsequent workers provided additional records from western Angola (Bengo Province: Parker 1934; Huambo and Huíla provinces: Monard 1938; Benguela Province: Monard 1938, Helmich 1957) and northeastern Angola (Lunda-Sul and Moxico provinces: Laurent 1964; Ruas 1996), all of which were reported as *B. mossambicus*. In a second review of the same material, Ruas (2002) revised her previous conclusions and referred the specimens from Moxico Province to the “*Breviceps mossambicus-adspersus* complex” (sensu Poynton 1982; Poynton and Broadley 1985), noting genetic data were needed to resolve their taxonomy. This species complex has been suggested to have a broad hybridization zone across southern Africa (Poynton 1982), and Angolan *Breviceps* were noted to share aspects of coloration with both *B. mossambicus* and *B. adspersus*, yet were distinct from *B. poweri* Parker, 1934 from the Zambezi Basin (Poynton and Broadley 1985). More recent synopses of Angolan material have either referred historical material to B. cf. adspersus (Baptista et al. 2019) or simply as *B.* sp. in recognition of the taxonomic uncertainties for these populations (Marques et al. 2018; Ceríaco et al. 2020).

**Figure 1.**
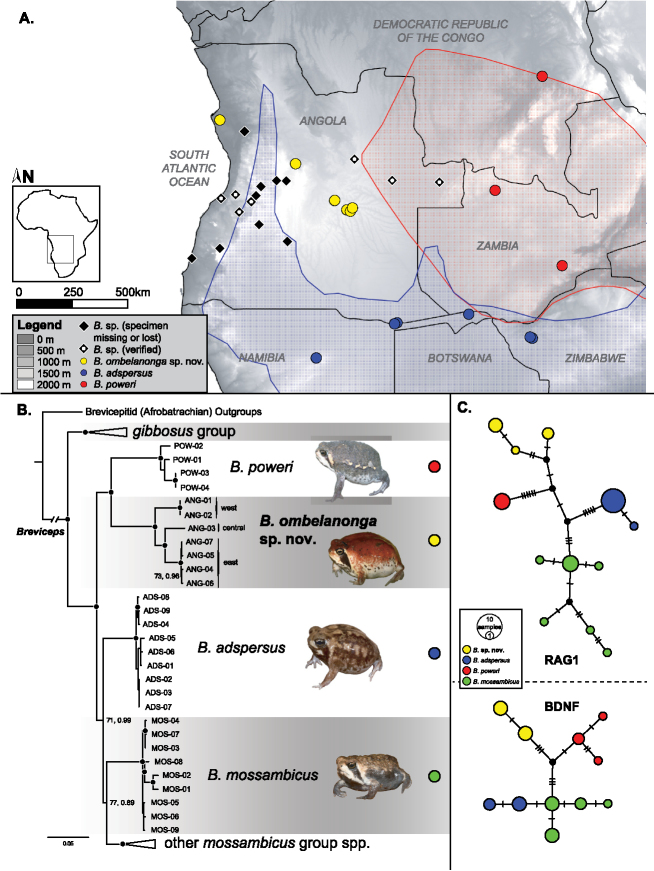
Geographic distribution and phylogenetic relationships of *Breviceps* spp. included in this study. **A** Map of Angola and surrounding countries with all known *Breviceps* spp. sampling localities indicated on legend. The proposed distributions of *B. adspersus* and *B. poweri* (blue and red polygons, respectively) are from IUCN (2013a, b), but should be considered tentative and worthy of reevaluation in light of recent studies. Furthermore, *B. mossambicus* is not mapped as no samples of certain identification occur west of Malawi (see Nielsen et al. 2018). **B** Multi-locus phylogeny of *Breviceps*, with select clades collapsed that are not relevant directly to the *B. mossambicus* group. The backbone is from the likelihood analysis, although Bayesian analyses produced a nearly identical topology (with any topological differences subtended by poor support). A black dot at each node indicates high support (e.g., Bayesian posterior probability > 0.95, Maximum Likelihood bootstrap > 90), while values below that cutoff are indicated for deep nodes only. Tapered bars to the right of voucher IDs indicate from which Angolan locality they were collected. **C** Median-joining networks for the two nuclear loci indicating a lack of shared haplotypes between candidate and recognized species. Hash marks indicate unique sequence differences between lineages, and black circles are hypothetical intermediate haplotypes.

A recent phylogenetic study of *Breviceps* (Nielsen et al. 2018), while lacking Angolan material, confirmed the presence of *B. poweri* in northwestern Zambia, as well as nomintotypical *B. adspersus* within 3 km of the Angolan border in Namibia (Fig. 1A). This suggests that both might also occur in Angola (Marques et al. 2018), although the evidence for *B. poweri* is based mainly on tertiary references (see Channing and Rödel 2019). Based solely on external morphology, Ceríaco and Marques (2018) recently identified specimens from Moxico Province, in eastern Angola, as *B. poweri*; these are the same specimens previously identified by Ruas (1996, 2002) as *B. mossambicus* and *B. mossambicus-adspersus*, respectively. While *B. mossambicus* has been historically listed as part of the Angolan anuran fauna, recent genetic analyses have so far only confirmed populations from Mozambique as corresponding to this name (Nielsen et al. 2018). Due to substantial morphological similarity, scarcity of genetic sampling, and potential for hybridization among *B. mossambicus*, *B. poweri*, and *B. adspersus* (Poynton 1964, 1982; Poynton and Broadley 1985; Minter et al. 2017), taxonomic identification of any historical Angolan material should therefore be considered tentative at best.

Angola’s long civil war, which lasted from 1975 to 2002, effectively stifled biological exploration and discovery (for additional summary, see Marques et al. 2018). Recent surveys, many by authors of this manuscript, have produced the only contemporary records of Angola’s herpetofauna (e.g., Ceríaco et al. 2014, 2016, 2018; Conradie et al. 2016; Heinicke et al. 2017; Marques et al. 2018; Baptista et al. 2019; Butler et al. 2019; Ernst et al. 2020), including the only recent records of *Breviceps* in Angola. The nearest samples with confident identifications and associated genetic data are at least 600 km away (i.e., *B. adspersus* in Namibia and *B. poweri* in Zambia; Nielsen et al. 2018). Here we analyze these recently collected Angolan *Breviceps* in a phylogenetic framework and assess their taxonomic status, resulting in the description of a new species so far known only from Angola.

## Materials and methods

### Species concept

We consider species as units of separately evolving metapopulation lineages, following the conceptual framework developed by Simpson (1951, 1961), Wiley (1978), and de Queiroz (2007).

### Sampling

Between 2016 and 2019, specimens referable to the genus *Breviceps* were collected from three main localities within Angola (Fig. 1A; Table 1). Animals were euthanized via immersion in or injection of MS-222 (tricaine methanesulfonate) soon after capture (Conroy et al. 2009). Tissue samples (liver) were removed postmortem and preserved in 95% ethanol for genetic analysis. Specimens were formalin-fixed for 48 hours and then transferred to 70% ethanol for long-term storage in the herpetological collections of the Florida Museum of Natural History (FLMNH), the Museu de História Natural e da Ciência da Universidade do Porto, Portugal (MHNCUP), South African Institute for Aquatic Biodiversity (SAIAB), and the Port Elizabeth Museum, South Africa (PEM). Besides the newly collected material, historical specimens housed in the collections of the Museum of Comparative Zoology at Harvard University, USA (MCZ), Musée d’Histoire Naturelle de La Chaux-de-Fonds (MHNC), the Natural History Museum of London, United Kingdom (NHMUK) the Zoologische Staatssammlung München, Germany (ZSM), the Instituto de Investigação Científica Tropical, Portugal (IICT), and the Museu Regional do Dundo, Angola (MD) were also consulted (see Appendix 1).

**Table 1.** Sampling information including specimens/field IDs (Museum Abbreviations: MCZ, Museum of Comparative Zoology, Harvard University, USA; MHNCUP, Natural History and Science Museum of the University of Porto, Portugal; MVZ, Museum of Vertebrate Zoology; PEM, Port Elizabeth Museum, South Africa; SAIAB, South African Institute for Aquatic Biodiversity, South Africa), GPS coordinates, and GenBank accession details for the samples included in our analyses.

species	Tree ID	Specimen ID	Field ID	Latitude and Longitude	Country	Locality	RAG1	BDNF	12S	16S
*Breviceps ombelanonga* sp. nov.	ANG-01	UF Herp 187172	MCZ A-36476	-9.183833, 13.371472	ANG	Kawa Camp (1 km S of the Kwanza R.), Kissama NP, Luanda Prov.	MT944215	MT944224	MT944230	MT944241
	ANG-02	UF Herp 187173	MCZ A-36495	-9.183833, 13.371472	ANG	Kawa Camp (1 km S of the Kwanza R.), Kissama NP, Luanda Prov.	MT944216	MT944225	MT944231	MT944242
	ANG-03	MHNCUP_ANF 0320	AMB11736	-11.083845, 16.667410	ANG	Embala Seque (14 km N of Cassumbi village), Bie Province	MT944217	MT944226	MT944232	MT944243
	ANG-04	PEM A12537	WC-3924	-12.689351, 18.360115	ANG	Cuito River source lake, Moxico Province	MT944218	MT944227	MT944233	MT944244
	ANG-05	PEM A12800	WC-4591	-13.089343, 18.894850	ANG	Cuanavale River source lake, Moxico Province	MT944219	MT944228	MT944234	MT944245
	ANG-06	PEM A12787	WC-4756	-13.135440, 19.043970	ANG	Quembo River source lake, Moxico Province	MT944220	MT944229	MT944235	MT944246
	ANG-07	PEM A12770	WC-4827	-13.003340, 19.135640	ANG	Cuando River source, Moxico Province	–	–	MT944236	MT944247
*B. adspersus*	ADS-01	MCZ A-137796	AMB8318	-22.708056, 29.528333	RSA	Farm Celine, Limpopo	MT944221	–	MT944237	MT944248
	ADS-02	MCZ Herp A-148603	MCZ-FS-A27931	-18.670972, 26.953472	ZIM	Hwange	MT944222	–	MT944238	MT944249
	ADS-03	MCZ Herp A-148653	MCZ-FS-A28024	18.628793, 26.872087	ZIM	Miombo Safari Camp	MT944223	–	MT944239	MT944250
	ADS-04	MCZ Herp A-149504	MCZ-FS-A28774	-19.528500, 17.564167	NAM	Farm Ohange, Otjozondjupa	–	–	MT944240	MT944251
	ADS-05	–	SVN 766	-23.731926, 27.579803	RSA	Ellisras	MH340062	MH340138	MH340291	MH340369
	ADS-06	–	SVN 768	-23.731926, 27.579803	RSA	Ellisras	MH340063	MH340139	MH340292	MH340370
	ADS-07	MCZ Herp A-148557	AMB7963	-17.623556, 24.199583	NAM	Katima Mulilo	MH340064	MH340140	MH340293	MH340371
	ADS-08	–	AMB7972	-18.000000, 21.070000	NAM	Caprivi	MH340065	MH340141	MH340294	MH340372
	ADS-09	MCZ Herp A-148563	AMB7980	-18.035500, 20.971528	NAM	Caprivi	MH340066	MH340142	MH340295	MH340373
*B. mossambicus*	MOS-01	MVZ:Herp:265910	DMP 344	-15.463942, 36.977847	MOZ	Gurue	MH340075	MH340151	MH340304	MH340382
	MOS-02	MCZ Herp A-137055	MCZ FS-A34284	-15.933333, 35.516667	MW	Mulanje	MH340076	MH340152	MH340305	MH340383
	MOS-03	SAIAB 88161.1	RB09-159	-15.030944, 40.740944	MOZ	Ila de Mozambique	MH340077	MH340153	MH340306	MH340384
	MOS-04	SAIAB 88161.2	RB09-179	-15.030944, 40.740944	MOZ	Ila de Mozambique	MH340078	MH340154	MH340307	MH340385
	MOS-05	SAIAB 88176.1	RB09-030	-12.963611, 40.529444	MOZ	Pemba	MH340079	MH340155	MH340308	MH340386
	MOS-06	SAIAB 88176.2	RB09-046	-12.963611, 40.529444	MOZ	Pemba	MH340080	MH340156	MH340309	MH340387
	MOS-07	SAIAB 88586	RB10-A097	-15.030722, 40.741222	MOZ	Nampula	MH340081	MH340157	MH340310	MH340388
	MOS-08	PEM A14008	NIMB 112	-13.308060, 35.244114	MOZ	Lichinga	MH340082	MH340158	MH340311	MH340389
	MOS-09	PEM A11021	Syran 12	-13.288667, 38.681528	MOZ	Balama	MH340083	MH340159	MH340312	MH340390
*B. poweri*	POW-01	–	ELI 325	-7.277700, 27.389800	DRC	Manono	MH340084	MH340160	MH340313	MH340391
	POW-02	SAIAB 98182	JWH10-A114	-12.237778, 25.341944	ZAM	Kalumbila	MH340085	MH340161	MH340314	MH340392
	POW-03	SAIAB 98788.1	RB10-F003	-15.510278, 28.260528	ZAM	Lusaka	MH340086	MH340162	MH340315	MH340393
	POW-04	SAIAB 98788.1	RB10-F012	-15.510278, 28.260528	ZAM	Lusaka	MH340087	MH340163	MH340316	MH340394

We amplified partial sequences of two mitochondrial (12S and 16S ribosomal rRNA genes) and two nuclear loci (recombination activating protein 1, RAG1; brain derived neurotrophic factor, *BDNF*) using the PCR primers and cycling conditions outlined in Nielsen et al. (2018). PCR success was evaluated via 1.5% agarose gel electrophoresis, then amplicons were sent to GeneWiz or the University of Michigan sequencing core for Sanger sequencing. We then assembled and quality trimmed raw sequences using Geneious v.8 (Biomatters; ​http://www.geneious.com). Sequences were submitted to GenBank (Table 1). Uncorrected mean pairwise sequence divergence (*p*) values were calculated for both 12S and 16S (Table 2) using MEGA v.6.0 (Tamura et al. 2013).

**Table 2.** Uncorrected mean pairwise 12S and 16S mitochondrial sequence differences between ingroup *Breviceps* sequence pairs (above/below the diagonal, respectively) and within species (along the diagonal) conducted in MEGA.

**16S/12S**		**12S**			
		***B. ombelanonga* sp. nov.**	***B. adspersus***	***B. poweri***	***B. mossambicus***
**16S**	***B. ombelanonga* sp. nov.**	**0.04/0.03**	0.09	0.09	0.11
	***B. adspersus***	0.11	**0.01/0.01**	0.09	0.08
	***B. poweri***	0.12	0.11	**0.02/0.02**	0.09
	***B. mossambicus***	0.12	0.08	0.10	**0.01/0.01**

### Phylogenetics

Datasets (concatenated and partitioned by locus/codon) of all samples were analyzed using maximum likelihood (RAxML v.8.2; Stamatakis 2014) and Bayesian (MrBayes v.3.2; Ronquist et al. 2012) methods via the CIPRES Science Gateway 3.1 for online phylogenetic analysis (Miller et al. 2010; http://www.phylo.org/index.php/portal/). Maximum likelihood analyses were performed using the default settings for RAxML using the GTRGAMMA model of sequence evolution (Stamatakis 2006) and ceasing bootstrapping when extended majority rule bootstrapping criteria had been reached. An appropriate partitioning strategy and molecular models for Bayesian analyses were chosen using PartitionFinder 2 (Lanfear et al. 2017), which assessed all possible candidate positions (e.g., each codon in the nuclear DNA) using the Bayesian information criterion. The resulting partition scheme is as follows: subset 1 (RAG1pos2, RAG1pos1) K80+G; subset 2 (RAG1pos3, BDNFpos3) K80+G; subset 3 (BDNFpos1, BDNFpos2) JC; and subset 4 (12S, 16S) GTR+I+G. Final Bayesian analyses ran for 100 million generations with four independent chains, and were sampled every 100,000 generations. We checked for stationarity using Tracer v.1.6 (Rambaut et al. 2018), after which a 25% burn-in was removed, leaving 750 trees for posterior analysis. For comparison with tree-based methods and in order to view gene tree (haplotype) relationships among the ingroup, median joining networks (MJN; Bandelt et al. 1999) for each nuDNA locus were constructed using PopART (http://popart.otago.ac.nz).

### Morphology

Specimens were measured to the nearest 0.1 mm using digital calipers under a dissecting stereomicroscope for the following 24 morphological characters as defined by Watters et al. (2016): snout-vent length (SVL, from the tip of the snout to the vent), snout-urostyle length (SUL, from the tip of the snout to the posterior end of the urostyle), head length (HL, from the posterior of the jaws to the tip of the snout), snout length (ES, from the tip of the snout to the anterior corner of the eye), nostril-ocular distance (NOD, from anterior corner of the eye to the posterior margin of the nostril), eye diameter (ED, horizontally from the anterior to posterior corner of the eye), nostril-upper lip distance (NLD, medial margin of nostril to ventral margin of upper lip), eye-upper lip distance (ELD, lower margin of eye to margin of upper lip), internarial distance (IND, between the inner margins of the nostrils), mouth width (MW, between the corners of the mouth), head width (HW, at the widest point; i.e, angle at the jaws), forearm length (EF3, elbow to base of digit 3), length of manual digit I (F1L, from distal end of digit to proximal base of most proximal subarticular tubercle), length of manual digit II (F2L, to proximal subarticular tubercle), length of manual digit III (F3L, to proximal subarticular tubercle), length of manual digit IV (F4L, to proximal subarticular tubercle), thigh length (THL, from vent to knee), crus length (CL, distance from the outer surface of the flexed knee to the heel/tibiotarsal inflection), length of pedal digit I (T1L, to distal margin of metatarsal tubercle), length of pedal digit III (T3L, to proximal subarticular tubercle), length of pedal digit IV (T4L, to proximal subarticular tubercle), foot length (FL, from the base of the inner metatarsal tubercle to the tip of pedal digit IV), length of pedal digit V (T5L, to distal margin of metatarsal tubercle), outer metatarsal tubercle length (OMTL), and inner metatarsal tubercle length (IMTL) when separate from OMTL. All measurements were taken on the right side of the body for consistency. A subset of ten measurements (HL, HW, ED, ES, IOD, IND, THL, CL, FL, and F3L) was taken from specimens of *B. adspersus* (n = 24), *B. mossambicus* (n = 9), *B. poweri* (n = 8), and the putative new Angolan species (n = 6) and checked for normality using a Shapiro-Wilks test (see Appendix 1, Suppl. material 1: Table S1). In order to avoid potential species misidentifications, specimens used in the comparative morphological analyses were derived from localities within the core geographic range of each species, as supported by the phylogenetic results of Nielsen et al. 2018. All were examined to confirm the presence of traits diagnostic for *B. adspersus*, *B. mossambicus*, or *B. poweri*, respectively. All measurements were corrected for body size via a generalized least squares linear regression on SVL using the *gls* function in R {nlme}. The residuals were then analyzed using the *prcom* (Principal Components Analysis; PCA) function in R {stats}. The components accounting for 75% of the cumulative variance were retrieved from the analysis. The relationship in morphospace between the putative new species and closely related *Breviceps* species was evaluated by plotting principal component (PC) scores.

### Advertisement calls

Advertisement calls were recorded in the field using an Samsung Galaxy Note 3 cellphone at a sampling rate of 44100 kHz, and analyzed using Sound Ruler Acoustic Analysis v.0.9.6.0 using default settings (Gridi-Papp 2007) and graphical presentations of calls were produced with the R package *seewave* (Sueur et al. 2008). Only a single male call was recorded from the Cuanavale River source lake (PEM A12800) on 24 October 2016. The call was compared to that of *B. mossambicus* and *B. poweri* from Ribaue, Mozambique, and to other published call data (Minter 1997, 2003). We further compared our call to that of *B. adspersus* provided by Du Preez and Carruthers (2017). The small number of calls did not allow for statistical analysis but the following standard measurements were taken: call duration, call interval, number of pulses per call, and dominant frequency in kilohertz (kHz).

### Nomenclatural acts

The electronic edition of this article conforms to the requirements of the amended International Code of Zoological Nomenclature (ICZN), and hence the new names contained herein are available under that Code from the electronic edition of this article. This published work and the nomenclatural acts it contains have been registered in ZooBank, the online registration system for the ICZN. The ZooBank LSIDs (Life Science Identifiers) can be resolved and the associated information viewed through any standard web browser by appending the LSID to the prefix “http://zoobank.org/”. The LSID for this publication is: http://zoobank.org/References/2043280A-1591-4D51-ACE3-F9015F170890. The electronic edition of this work was published in a journal with an ISSN, and has been archived and is available from the following digital repositories: PubMed Central, LOCKSS.

## Results

### Phylogenetics

Our concatenated, multi-locus dataset was 1,852 bp long, of which 390 characters were parsimony informative. Phylogenetic analyses resulted in a well-supported species-level phylogeny and high support that *Breviceps* is monophyletic (bootstrap [bs] 100, posterior probability [pp] 1.0; Fig. 1B). All Angolan samples were recovered as monophyletic with high support (bs 100, pp 1.0), sister to *B. poweri* (bs 86, pp 1.0), and thus embedded within the *B. mossambicus* group (bs 99, pp 1.0). We also failed to recover any nuclear haplotype sharing among taxa (Fig. 1C). We recovered high genetic divergence (≥ 9–11% 12S/16S uncorrected *p*-distances; Table 2) between the Angolan material and the three most closely related (and potentially sympatric and/or morphologically similar) taxa, *B. adspersus*, *B. mossambicus*, and *B. poweri*, as well as substantial intraspecific diversity (3–4% 12S+16S uncorrected *p*-distances). The values are comparable with, or exceed other species level differences within recognized species of *Breviceps* (see Nielsen et al. 2018).

### Morphology

**Figure 2.**
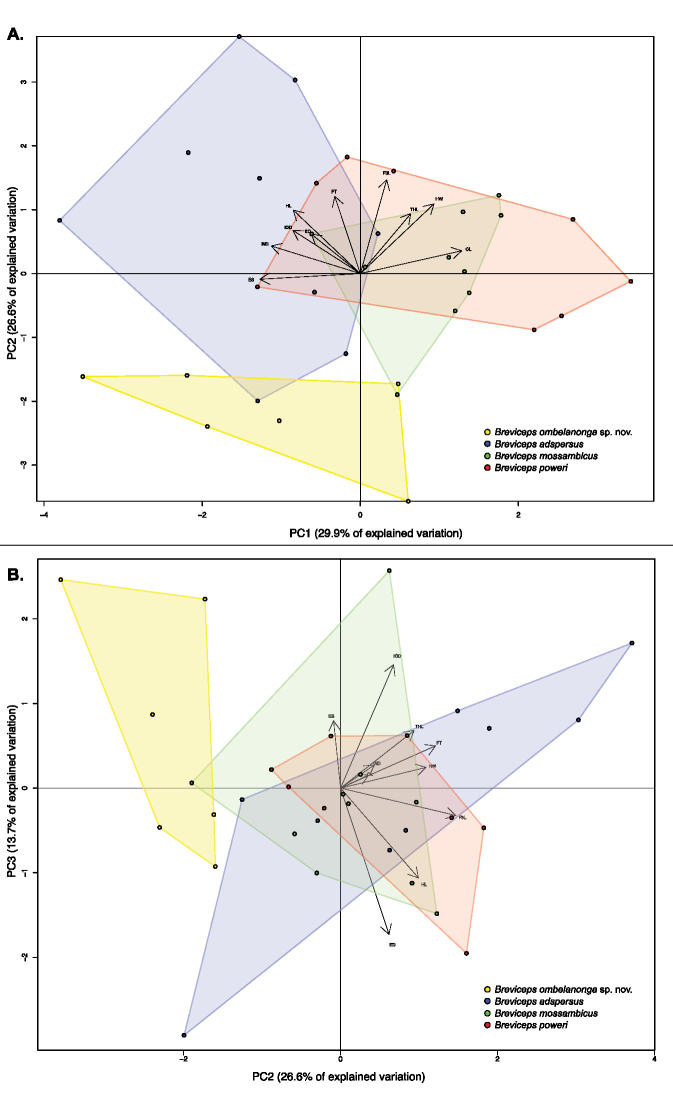
PCA plots of ten size-corrected morphological characters from specimens of *B. adspersus* (n = 24), *B. mossambicus* (n = 9), *B. poweri* (n = 8), and the putative new Angolan species (n = 6) (Suppl. material 1: Table S1), illustrating the PC1 and PC2 (**A**) and PC2 and PC3 (**B**) axes of variation, which combined represent ~ 70% of the total variation (Table 4).

Mensural and meristic data are presented in Table 3. The first four principal components account for 78.9% of the variation in the data (Table 4). The first principal component loads strongly on the measurements of head shape and limb length, including strong negative loadings on head length and snout length, and positive loadings for crus length, but does not differentiate the putative new species from Angola from its close relatives (Fig. 2). The second principal component axis loads strongly and positively on measurements of head width, thigh length, the lengths of the third manual digit and foot, and distinguishes the new species from other species due to its more narrow head, shorter thigh, and shorter third manual digit and foot. The third principal component has a strong negative loading on the diameter of the eye and a strong positive loading on distance between orbits, but the new species is not distinguished from other species on this axis.

**Table 3.** Measurements (mm) of type series.

	UF Herp 187172	UF Herp 187173	MHNCUP_ANF 0320	PEM A12800	PEM A12537	PEM A12787	PEM A12770	SAIAB 204537	Average	SD
SVL	30.5	27.5	24.6	25.4	18.3	26.6	30.1	26.5	**26.2**	**3.80**
SUL	26.3	24.9	–	23	17.6	23.2	29	25.2	**24.2**	**3.53**
HL	7.2	7.3	7.3	10.8	6.4	9.6	12.7	9.3	**8.8**	**2.17**
ES	3.2	2.8	2.9	3.1	2.2	2.7	3.3	2.5	**2.8**	**0.37**
NOD	2.0	1.7	1.6	2.1	2.5	1.7	2.2	2.1	**2.0**	**0.30**
ED	2.7	2.8	3.2	3.4	1.3	3.1	3.6	2.8	**2.9**	**0.70**
NLD	1.7	1.7	1.3	1.4	1	1.2	1.8	1.3	**1.4**	**0.28**
ELD	1.7	1.3	1.7	1.8	1.4	2.1	2.3	1.9	**1.8**	**0.33**
IND	1.9	1.9	2	1.6	1.4	1.8	2.2	2	**1.9**	**0.25**
MW	7.2	6.8	5.1	6.9	4.7	6.3	7.9	7	**6.5**	**1.08**
EAD	9.1	9.3	4.1	4.5	3.6	4.3	5.3	–	**5.7**	**2.42**
F1L	1.7	1.5	1.5	2.7	1.7	2.7	2.5	2.8	**2.1**	**0.59**
F2L	1.9	1.9	1.7	3.6	1.8	3.2	2.6	2.7	**2.4**	**0.71**
F3L	2.9	3.0	2.2	3.9	2.6	3.9	3.6	3.7	**3.2**	**0.64**
F4L	1.2	1.3	1.2	1.8	0.9	2	1.5	1.4	**1.4**	**0.35**
T1L	1.0	1.1	1	1.4	0.6	1.5	1.6	1.2	**1.2**	**0.32**
T3L	1.8	1.9	2.9	2.4	2	2.7	1.5	2.7	**2.2**	**0.51**
T4L	4.4	4.2	4.6	4.1	3.2	5	4.9	4.6	**4.4**	**0.57**
FT	10.9	10.4	8	8.9	6	10.3	10.5	9.7	**9.3**	**1.65**
T5L	4.0	5.0	0.8	4	3.2	4.9	5.3	4.7	**4.0**	**1.46**
MTL	1.0	1.0	1.2	2.3	1.7	2.3	–	–	**1.6**	**0.61**
IMTL	3.0	2.9	3	3.2	2.3	3.4	3.6	3.7	**3.1**	**0.45**
TIB	8.5	8.3	–	6.4	4.8	7.4	8.8	8.1	**7.5**	**1.43**

### Advertisement calls

**Figure 3.**
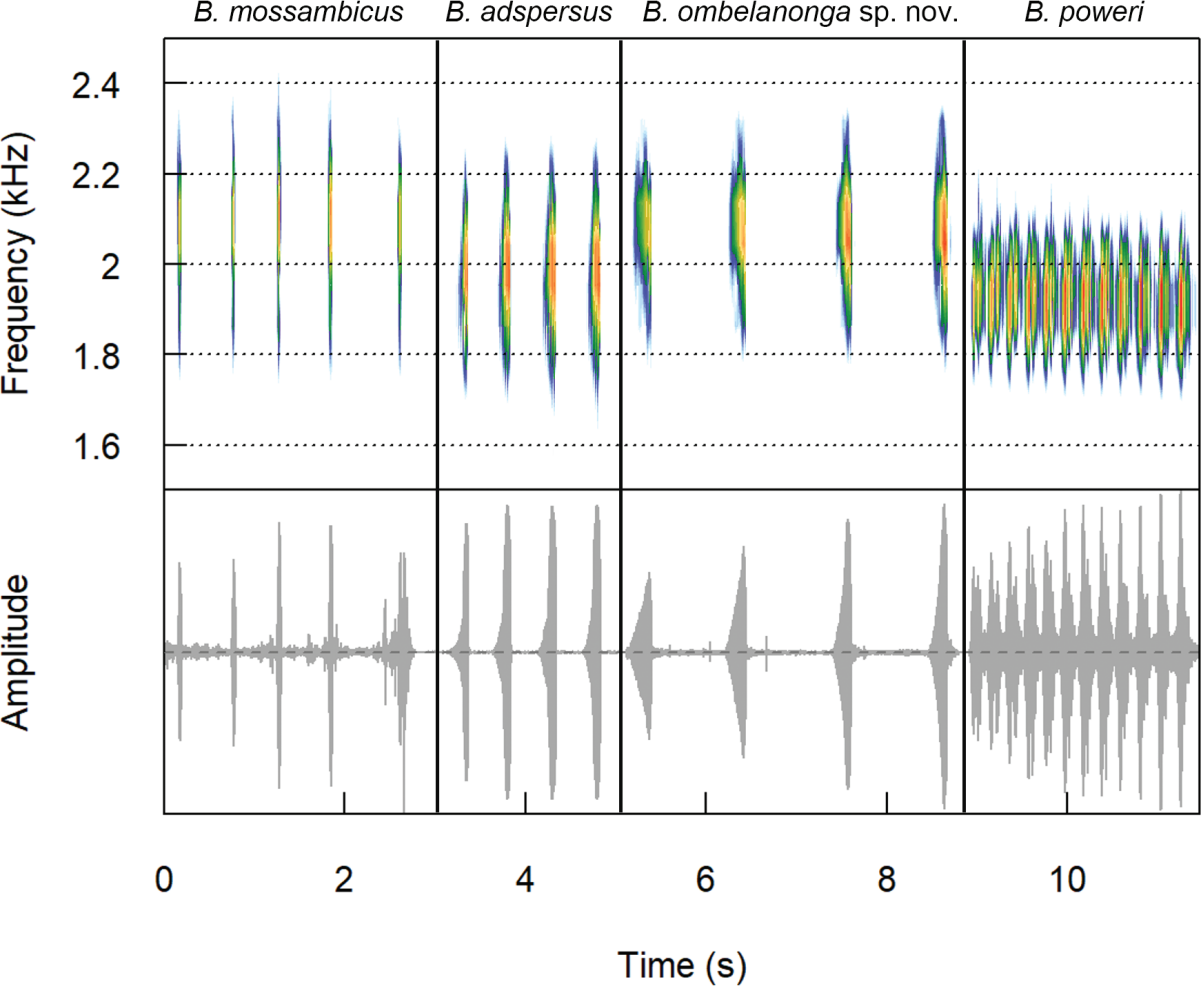
Spectrograms and oscillograms showing a series of notes of the putatively novel Angolan *Breviceps* taxon compared to three closely related congeners.

The advertisement call of the eastern population is pulsed, has a call duration of 0.175 ± 0.083 s, with relatively long intervals between consecutive calls (0.996 ± 0.133 s), a high number of pulses per call (28–34; Table 4, Fig. 3), and a dominant call frequency of 2156 Hz. It most resembles the whistle-like call of *B. adspersus* (call duration: 0.196 ± 0.047 s; interval between consecutive calls: 0.745 ± 0.636 s; pulses per call: 14–31), yet differs from the ‘chirp’-like call of *B. mossambicus* (call duration: 0.500 ± 0.070 s; interval between consecutive calls: 0.710 ± 0.168 s; pulses per call: 7–31) and the tonal, rapid call of *B. poweri* (pulses per call: 7–31; dominant call frequency: 1557 –1903 Hz). Because ambient temperature was not documented when the call was recorded, these results carry some uncertainty.

**Table 4.** Principal components analysis (PCA) loadings based on 10 size corrected morphological characters (head length, HL; head width, HW; eye diameter, ED; snout length, ES; interorbital distance, IOD; internarial distance, IND; thigh length, THL; crus length, CL; foot length, FL; and length of manual digit III, F3L).

	**PC1**	**PC2**	**PC3**	**PC4**	**SShapiro-Wilks test**
Proportion of Variance	29.92	26.57	13.70	8.67	
Cumulative Proportion	29.92	56.49	70.19	78.86	
Loadings					
Head Length (HL)	-0.3025245	0.35446298	-0.3789378	0.02808779	W = 0.943, p = 0.083
Head Width (HW)	0.33281314	0.38971121	0.08705045	0.33211061	W = 0.956, p = 0.205
Eye diameter (ED)	-0.2210569	0.22060777	-0.6170786	-0.1165699	W = 0.963, p = 0.312
Snout length (ES)	-0.4530121	-0.0316756	0.28394189	-0.1665634	W = 0.973, p = 0.567
Interorbital distance (IOD)	-0.3043034	0.24152256	0.52046607	0.21274977	W = 0.959, p = 0.240
Internarial distance (IND)	-0.4018886	0.1543163	0.09759718	0.52889612	W = 0.965, p = 0.360
Thigh length (THL)	0.2280386	0.3347664	0.24257868	-0.4343642	W = 0.965, p = 0.360
Crus length (CL)	0.45829394	0.12745601	0.05499745	0.20448849	W = 0.975, p = 0.636
Pes length (FT)	-0.1152479	0.43230304	0.17838021	-0.5130485	W = 0.900, p = 0.005
Manual digit III length (F3L)	0.11914774	0.52484652	-0.115415	0.16994577	W = 0.989, p = 0.978

## Systematics

Our phylogenetic analyses indicate that sampled individuals from Angola form a clade that is genealogically exclusive from other described species of *Breviceps* (Fig. 1). These populations are morphologically diagnosable from other closely related taxa, specifically possessing distinct coloration and pattern that differ from the sister taxon, *B. poweri*. A PCA of mensural and meristic data indicates that the Angolan individuals fall within a unique region of morphospace, with a relatively narrower head, shorter thigh, and shorter manual digit III in comparison to closely related species. Lastly, there are distinct acoustic differences associated with the male nuptial call. Thus, we here describe these populations as a new species.

### Breviceps ombelanonga sp. nov.

Taxon classificationAnimaliaAnuraBrevicipitidae

978CF7A2-1BAB-5281-9133-7943C913494E

http://zoobank.org/E3815018-4176-4073-92B8-E65274D354FB

**Figure 4.**
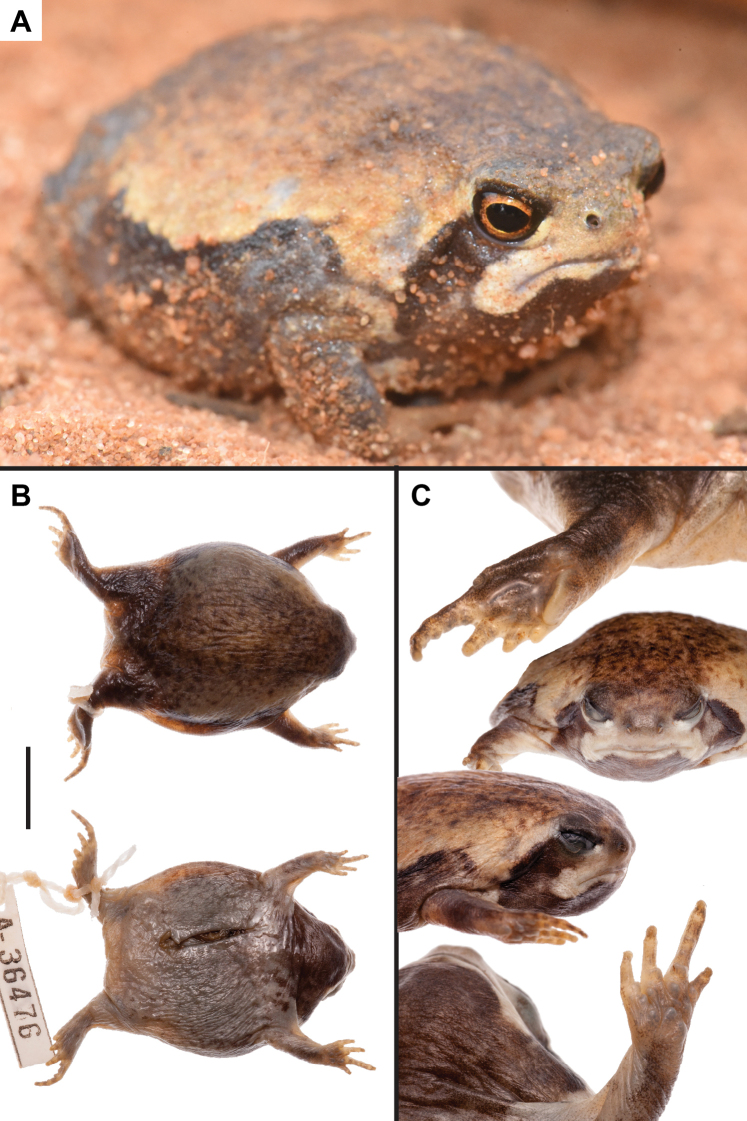
*Breviceps ombelanonga* sp. nov. holotype male (UF Herp 187172): **A** in life photo **B** dorsal and ventral aspects **C** additional views of the holotype, including the left pes, frontal, right lateral, and left manus and mental. Scale bar: 10 mm. Photographs by J. Cavagnaro (**A**) and SVN (**B, C**).

**Figure 5.**
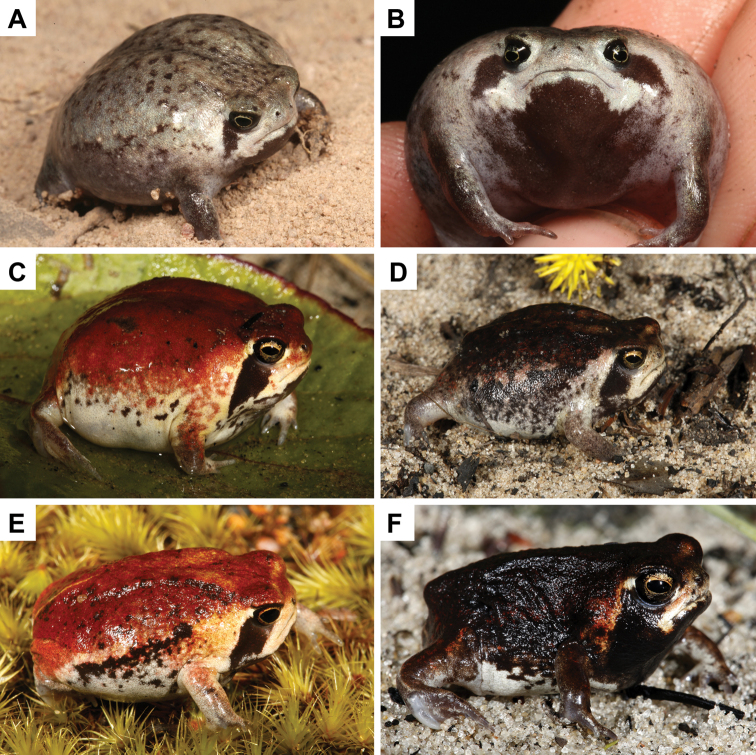
Variation in color and pattern within living paratypes of *B. ombelanonga* sp. nov.: **A, B** sub-adult (of unknown sex) from Embala Seque (14 km N of Cassumbi village), Bié Province (MHNCUP_ANF 0320) **C** juvenile male, Cuito River source lake, Moxico Province (PEM A12537) **D** adult female, Cuando River source, Moxico Province (PEM A12770) **E** adult male, Quembo River source lake, Moxico Province (PEM A12787) **F** adult male, Cuanavale River source lake, Moxico Province (PEM A12800). Photographs by LMPC (**A, B**) and WC (**C–F**).

**Figure 6.**
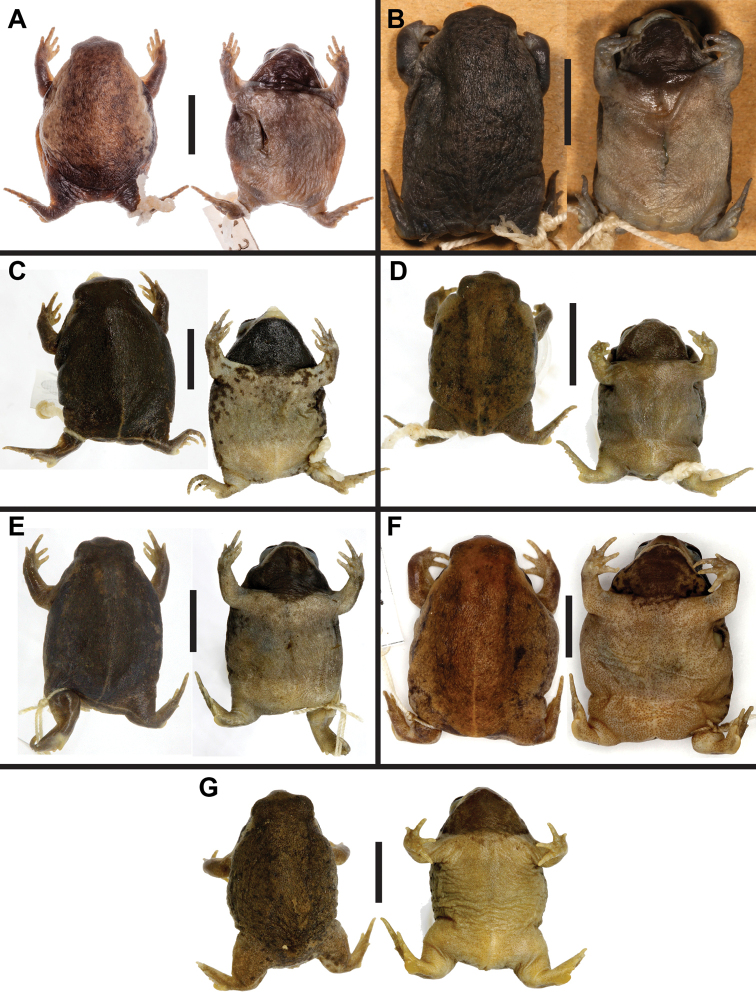
Variation in color and pattern within preserved paratypes of *B. ombelanonga* sp. nov.: **A** an adult male from Kawa Camp Headquarters, Luanda Province (UF Herp 187173) **B** sub-adult (of unknown sex) from Embala Seque, Bié Province (MHNCUPANF 320) **C** adult male from Cuanavale River source lake, Moxico Province (PEM A12800) **D** juvenile male from Cuito River source lake, Moxico Province (PEM A12537) **E** adult male from Quembo River source lake, Moxico Province (PEM A12787) **F** adult female from Cuando River source, Moxico Province (PEM A12770); and **G** adult male from Quembo River source lake, Moxico Province (SAIAB 204537). Photographs by SVN (**A**), LMPC (**B**), and WC (**C–G**).

**Figure 7.**
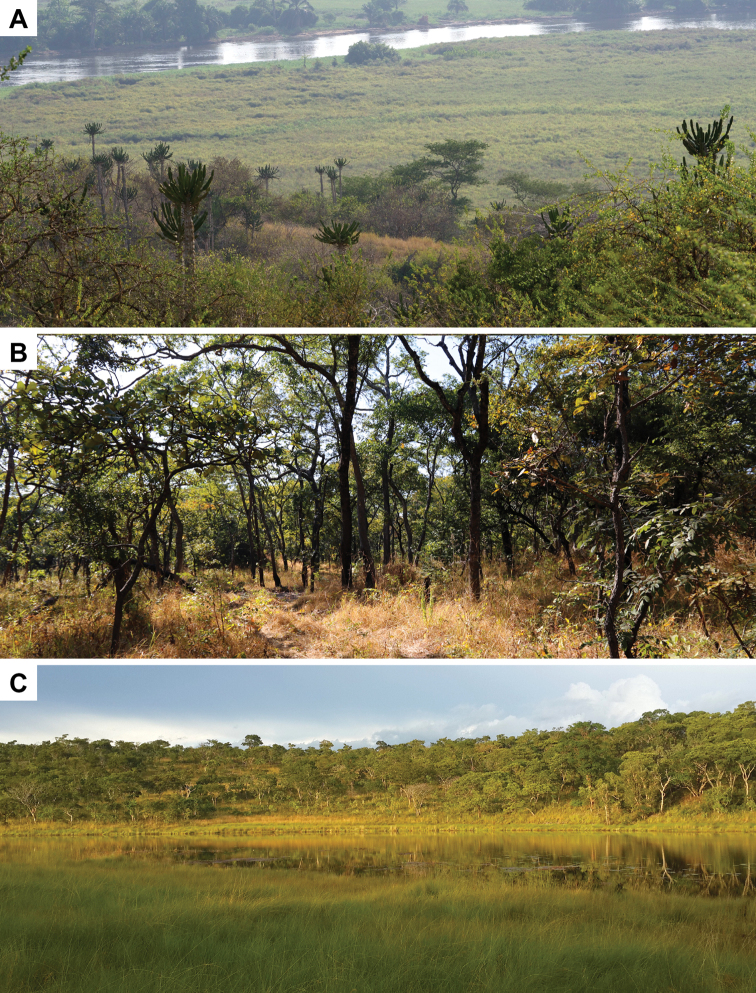
Photos of typical habitat of *B. ombelanonga* sp. nov.: **A** a view of the Kwanza River and bordering savannah, near the type locality, in Kissama National Park, Luanda Province **B** savannah near Embala Seque (14 km N of Cassumbi village), Bié Province **C** Cuanavale River source lake and associated miombo savannah woodland. Photographs by LMPC (**A, B**) and WC (**C**).

Figs 4 , 5 , 6 , 7 Suggested common names: Angolan Rain Frog (English), Sapinho das Chuvas de Angola (Português).

#### Chresonymy.^1^

*Breviceps gibbosus*: Bocage (1870: 68).

*Breviceps gibbosus*: Bocage (1873: 227).

*Breviceps mossambicus*: Bocage (1895: 182); Parker (1934: 194); Monard (1937: 29, 1938: 56); Laurent (1964: 156); Cei (1977: 17, 18); Ruas (1996: 23).

*Rana mossambicus*: Hellmich (1957: 30).

*Breviceps* “*mossambicus-adspersus*” complex: Poynton (1982: 67); Ruas (2002: 142).

*Breviceps adspersus* [part]: Poynton and Broadley (1985: 52).

*Breviceps* sp.: Marques et al. (2018: 81); Ceríaco et al. (2020: 63).

Breviceps cf. adspersus: Baptista et al. (2019: 270).

#### Material examined.

***Holotype*.** UF Herp 187172 (field number MCZ A-36476), an adult male, Kawa Camp Headquarters, 1 km south of the Kwanza River, Kissama National Park (-9.183068, 13.369314, WGS-84, elevation 151 m above sea level), Luanda Province, Angola, collected by LMPC, Mariana P. Marques, Philip Pastor, and John Cavagnaro on 2 June 2016 at approx. 22:00. ***Paratypes*** (5 males, 1 female, 1 sex unknown) UF Herp 187173 (field number MCZ A-36495), an adult male, Kawa Camp Headquarters, 1 km south of the Kwanza River, Kissama National Park (-9.183068, 13.369314, WGS-84, elevation 151 m above sea level), Luanda Province, Angola, collected by LMPC, Mariana P. Marques, Philip Pastor, and John Cavagnaro on 8 June 2016; MHNCUP/ANF 320 (field number AMB 11736), sub-adult (sex unknown), Embala Seque, 14 km N of Cassumbi village (-11.083845, 16.66741), Bié Province, Angola, collected by LMPC, Mariana P. Marques, and Adam Ferguson on 16 June 2019; PEM A12800 (field number WC-4591), adult male, Cuanavale River source lake (-13.089343, 18.89485, 1396 m above sea level), Moxico Province, Angola, collected by Werner Conradie and Luke Verburgt on 24 October 2016; PEM A12537 (field number WC-3924), juvenile male, Cuito River source lake (-12.68935, 18.36012, 1435 m above sea level), Moxico Province, Angola, collected by Werner Conradie and Ninda Baptista on 18 February 2016 October; PEM A12787 (field number WC-4756), adult male, Quembo River source lake (-13.13544, 19.04397, 1375 m above sea level), Moxico Province, Angola collected by Werner Conradie on 11 November 2016; PEM A12770 (field number WC-4827), adult female, Cuando River source (-13.00334, 19.13564, 1364 m above sea level), Moxico Province, Angola, collected by Werner Conradie and James Harvey on 22 November 2016; SAIAB 204537 (field number Ang16-RB12), adult male, Quembo River source lake (-13.13583, 19.04528), Moxico Province, Angola, collected by Roger Bills on 9 November 2016.

#### Diagnosis.

A species referable to *Breviceps* due to the following characteristics (Poynton 1964; Minter et al. 2017): snout extremely abbreviated; mouth narrow and downturned near jaw joint; short limbs which, at rest in life, are held close to the body, not projecting beyond the body outline; digits I and V short or rudimentary; inner metatarsal tubercle well developed and notably longer than pedal digit III, narrowly separated from a prominent conical outer metatarsal tubercle. Additionally, the results of the molecular phylogenetic analyses support this species as embedded within the diversity of *Breviceps*, specifically within the *B. mossambicus* group (Fig. 1B). *Breviceps ombelanonga* can be diagnosed from other species of *Breviceps* and especially those in the *B. mossambicus* group by the combination of lacking a visible tympanum, males having a single, uniformly dark gular patch that is continuous with the mask extending from the eye, having generally smooth dorsal skin, lacking many small tubercles on the palmar surfaces (as in, e.g., *B. branchi* and *B. sylvestris*; FitzSimons 1930; Channing 2012), lacking pale spots along flanks and a pale patch above the vent (both present in *B. poweri*; Parker 1934; du Preez and Carruthers 2017), lacking short dark band below nares (as in *B. poweri*; du Preez and Carruthers 2017), lacking confluent inner and outer metatarsal tubercles, having a relatively narrower head, shorter thigh, and shorter manual digit III (Fig. 2; Table 4), and having an advertisement call with both a longer interval between consecutive calls and a higher average dominant frequency (Fig. 3).

The new species can be distinguished from other species of *Breviceps* occurring in the region by the following: pale paravertebral and dorsolateral patches are lacking, although a fine dorsolateral band may be present (versus no pale paravertebral or dorsolateral spots or patches in *B. mossambicus*; series of both paravertebral and dorsolateral pale spots and patches present in *B. adspersus*, a series of pale dorsolateral spots or patches present in *B. poweri*); no conspicuous light patch above vent (present in *B. poweri*); manual digit IV reaching approximately midway between the proximal and distal subarticular tubercles of manual digit III (versus nearly reaching distal subarticular tubercle of manual digit III in *B. mossambicus*; not reaching or barely passing the proximal subarticular tubercle of the manual digit III in *B. poweri*; similar to *B. adspersus* in usually not reaching the distal subarticular of manual digit III); gular region with a single uniformly dark patch (versus a pair of marbled to freckled patches in *B. adspersus*).

The advertisement call of the new species (Table 5, Fig. 3) can be differentiated from other potential Angolan congeners by its duration (0.175 ± 0.083 s; shorter than in *B. adspersus* 0.196 ± 0.047 s and *B. mossambicus*, 0.500 ± 0.070 s, and longer than in *B. poweri*, 0.140 ± 0.012 s), longer interval between consecutive calls (0.996 ± 0.133 s; *B. adspersus*, 0.745 ± 0.636 s; *B. mossambicus*, 0.710 ± 0.168 s; *B. poweri*, 0.743 ± 0.166 s), and a higher dominant frequency (2156 Hz; *B. adspersus*, 1742 ± 100 Hz; *B. mossambicus*, 1835 ± 107 Hz; *B. poweri*, 1728 ± 83 Hz). The number of pulses per call (28–34) are similar to *B. adspersus* (14–31), *B. mossambicus* (7–31), and *B. poweri* (10–74).

**Table 5.** Comparison of the main variables for the advertisement calls of *Breviceps ombelanonga* sp. nov., *Breviceps mossambicus*, *Breviceps adspersus* and *Breviceps poweri*. Comparative data taken from Minter (1997, 2003).

	*B. ombelanonga* sp. nov.		*B. adspersus*		*B. mossambicus*		*B. poweri*	
	avg ± sd	range	avg ± sd	range	avg ± sd	range	avg ± sd	range
**Call duration (s)**	0.175 ± 0.083	0.064–0.342	0.196 ± 0.047	0.077–0.293	0.500 ± 0.070	0.036–0.079	0.140 ± 0.012	0.111–0.160
**Call interval (s)**	0.996 ± 0.133	0.742–1.190	0.745 ± 0.636	0.363–0.745*	0.710 ± 0.168	0.396–1.17	0.743 ± 0.166	0.500–1.100
**No. of pulses/call**	30 ± 2.6	28–34	23 ± 3.3	14–31	9 ± 1.2	7–13	30 ± 16.3	10–74
**Dominant frequency (Hz)**	2156	na	1742 ± 100	1482–2179	1835 ± 107	1600–2193	1728 ± 83	1557–1903

#### Description of the holotype.

Adult male (SUL 30.5 mm), with globular body and well-developed short limbs with medialmost and lateralmost digits reduced (Fig. 4; Table 3); snout abbreviated, protruding and angular in lateral profile, blunt and rectangular in dorsal view; eyes projecting beyond profile of head in both dorsal and ventral views; pupils horizontally elliptical; nares small oval slits, directed horizontally and visible in dorsal and lateral views; mouth narrow and directed ventrally near jaw joint; choana largely obscured by maxillae in ventral view; well-developed gland at midline of palate between choana; tongue ovoid and filling floor of mouth, and lacking median papilla; single medial bony point on lower jaw at symphysis; tympana not distinguishable; teeth absent on premaxilla, maxilla, and vomer.

Skin of dorsum and head smooth, and weakly glandular with irregular folds; skin of ventrum smooth; skin folds overlying vent creating triangular shape.

Limbs short with digits I and V short or rudimentary; webbing absent on manus and pes; nuptial pads absent and adhesive glands not discernable; relative manual digit lengths when adpressed: III>II>I>IV; only tip of first pedal digit extending beyond fleshy webbing and sole; fourth (outer) manual digit reaches midway between the large tubercle at metacarpophalangeal joint and subarticular tubercle at most proximal interphalangeal joint; finger tips conical, not expanded; several small globular palmar tubercles; single subarticular tubercles present on pedal digits II, III, and IV; pedal digit V very short, falling short of most proximal subarticular tubercle of pedal digit IV; well-developed (though not keratinized) inner metatarsal tubercle visibly longer than pedal digit III, separated from conical outer metatarsal tubercle by deep cleft.

#### Coloration.

In life, dorsum of body mottled dark brown on pale tan base, transitioning to golden yellow on the lateral aspects, before stark transition to solid dark brown flanks with a dark boundary becoming paler ventrally (Fig. 4); limbs dark grayish brown dorsally; plantar and palmar surfaces pale grayish brown; subarticular, palmar, and inner and outer metatarsal tubercles pale gray; posterior dorsum dark gray-brown with scattered pale gray spots; bold facial mask composed of broad dark brown stripe running obliquely downwards, from margin of lower eyelid towards base of arm (but not attaining it) and joining dorsolateral aspect of gular patch, giving appearance of a large dark bib; region below nares generally same coloration as dorsal and lateral rostrum, and not more darkly pigmented; lower eyelid with white opaque patch at anterior margin; margins of mouth and lateral angle of mouth off-white to cream; gular patch uniformly dark anteriorly, becoming mottled posteriorly and merging with ventral coloration; pectoral region and ventrum creamy pale gray with scattered punctate gray dots sometimes coalescing into larger spots in the gular region and laterally; iris bright orange, scattered with dark brown flecks (dark brown in preservative), with black pupil (pale gray in preservative; no mid-vertebral line; faint pale line extending across posterior hindlimbs extending between heels.

In preservative, coloration is largely similar but more muted and overall darker (Fig. 4).

#### Measurements.

Measurements of the type series are shown in Table 3.

#### Variations.

All specimens resemble the holotype in the absence of a visible tympanum, and skin that is densely granular dorsally and laterally and smooth ventrally (Figs 5–6). The distal tip of manual digit IV reaches well past the proximal subarticular tubercle of manual digit III in all specimens. PEM A12770 have both manual digit II and III proximal subarticular tubercles divided. Inner and outer metatarsal tubercles not separated by a deep cleft in paratypes PEM A12800, PEM A12537, PEM A12787, PEM A12770 and SAIAB 204537.

Color and pattern in UF Herp 187173 is very similar to the holotype. Dorsum gray with scattered black spots (MHNCUP/ANF 320); red with scattered black blotches in two specimens (PEM A12537 and PEM A12770), dark brown to black with red spots and markings (PEM A12787 and PEM A12800), light brown with red spots and darker black blotches (SAIAB 204537). Interocular bar visible in all paratypes, except PEM A12537, PEM A12770 and SAIAB 204537. Light dorsolateral patches present in PEM A12878, absent in PEM A12770, dark black band present in PEM A12537. Mid-vertebral line present in most paratypes, but very faint in PEM A12537 and PEM A12770, and absent in SAIAB 204537 and MHNCUP/ANF 320. Heel-to-heel line present in all specimens, but faint in UF Herp 187173, PEM A12537, and PEM A12770. A broad, black stripe runs obliquely downwards from margin of lower eyelid towards base of arm, not reaching the shoulder in all specimens. Dark orbital band partly reaching the gular patch in all specimens, falling short in PEM A12770 (female). Anterior to the orbital bar, a broad white stripe runs down to angle of mouth and onto upper and lower lips in all individuals. Gular patch uniform dark brown to black in all paratypes, except PEM A12537 in which it is dark brown with scattered darker blotches. Pectoral region white, with scattered spots in all specimens. Ventrum white with scattered darker spots in all paratypes.

#### Advertisement call.

The following call description is based on a recording of a paratype male (PEM A12800) from the source lake of the Cuanavale River recorded on 24 October 2016 at 8:50 in the morning. Ambient temperature was not recorded. Frogs began calling during the daytime following heavy rains, and stopped after sunset. Call sites were among leaf litter in dense miombo woodland. The call can be described as a short whistle with a call duration of 0.064–0.342 seconds and call interval of 0.742–1.190 seconds. Each call consists of about 28–34 pulses and a dominant frequency of 2156 Hz (Table 4, Fig. 2). The small number of calls from a geographically restricted sample does not allow for further statistical analysis.

#### Distribution.

Based on our phylogenetic analysis, this species is currently confirmed from three widely separated localities and elevations ranging from near sea level to > 1400 m: i) Kissama National Park, on the outskirts of Angola’s capital city, Luanda, in coastal western Angola (Luanda Province); ii) central Angola (Bié Province); and iii) the source of the Cuanavale, Cuito, Cuando and Quembo rivers (Moxico Province). The identity of other known Angolan localities for *Breviceps* (black diamonds) remain uncertain without additional sampling and genetic data (Fig. 1, Appendix 1; see Marques et al. 2018).

#### Genetic divergence.

*Breviceps ombelanonga* differs from other species within the *B. mossambicus* group by net uncorrected mitochondrial *p*-distances of at least 9% (12S) and 11% (16S; Table 2), as well as unique nuclear haplotypes for both RAG1 and BDNF (Fig. 1B).

#### Habitat and natural history notes.

The preferred habitat for *B. ombelanonga* ranges from typical western Angolan savannah, with sandy soils and vegetation dominated by *Adansonia digitata*, *Euphorbia conspicua*, *Acacia welwitschii* and *Combretum* sp., together with a good grass coverage (Grandvaux-Barbosa 1970), to dense Angolan wet miombo woodland in the east (Fig. 7). The type series was collected after gentle rains, either by hand or in traps. The holotype was first observed feeding on small, unidentified ants (family Formicidae). No information is available on egg deposit sites and clutch sizes. One of us (WC) has discovered remains of *B. ombelanonga* in the stomach contents of two snake species, *Kladirostratus acutus* (Psammophiidae; PEM R23450) and *Causus bilineatus* (Viperidae; PEM R23321) from the Cuando and Cuito River sources, respectively.

#### Etymology.

The name *ombelanonga* is a derived combination of two words in Umbundu, a native Angolan language, for rain (*ombela*) and frog (*anonga*). The species epithet is used as an invariable noun in apposition to the generic name.

#### Conservation status.

Given that it appears widely distributed, we suggest that *B. ombelanonga* be included in the IUCN category of Least Concern. The type locality lies within Kissama National Park, which grants some legal protection from major habitat degradation and loss, though the park has recently experienced significant wildfires. Additionally, the paratype localities in southeastern Angola (visited during field activities related to the National Geographic Okavango Wilderness Project 2017) are relatively pristine and ecologically intact miombo savannah that comprise an area recently proposed for formal protection.

## Discussion

*Breviceps ombelanonga* sp. nov. represents a phylogenetically distinct evolutionary lineage that is an Angolan endemic apparently geographically isolated from its closest congeners (Fig. 1). It forms a clade with morphologically similar members of the *B. mossambicus* group but can be differentiated from its sister taxon, *B. poweri*, phylogenetically, morphologically, and acoustically (Figs 1–3). Unlike *B. poweri*, *B. ombelanonga* lacks pale spots along the flanks, a pale patch above the vent, and a short, dark band below the nares (Fig. 4). We also recovered high intraspecific genetic diversity among populations of *B. ombelanonga*, which for the most part exceeds the reported interspecific distances for some recently described *Breviceps* species (Minter 2003; Minter et al. 2017; see Nielsen et al. 2018). Given the limited morphological variation within the novel taxon (and the *B. mossambicus* group more broadly), we elected to conservatively consider these three disjunct populations as one taxon.

Further work is required to confirm the distributional range of *B. ombelanonga*, as well as whether it overlaps in distribution with either its sister taxon, *B. poweri*, or the more distantly related *B. adspersus*. Both occur in neighboring countries, *B. poweri* to the east/northeast (Zambia, Democratic Republic of Congo) and the *B. adspersus* to the south/southeast (Namibia, Botswana), and both have been suggested to occur in Angola (Ruas 2002; Marques et al. 2018; Baptista et al. 2019; Channing and Rödel 2019; Fig. 1A). Due to the amount of morphological similarity found among most members of the *B. mossambicus* group, identifying museum specimens to species is difficult without having genetic data with which to assign populations. Therefore, we have elected to leave the historical specimens from Angola as unassigned (see Appendix 1). Revisiting historical collection localities, or in some cases attempting to acquire ‘historical’ DNA sequence data from museum specimens, carries high priority and should help to illuminate the composition and distribution of Angola’s resident *Breviceps* species.

We are not the first to recognize the lack of morphological variation within members of this anuran clade, which has led to historical taxonomic confusion and invoking hybridization for specimens that failed to conform to often scant descriptions of the type specimens (Poynton 1964, 1982; Poynton and Broadley 1985; Minter et al. 2017). The only comprehensive molecular phylogenetic study to date failed to find support for hybridization (Nielsen et al. 2018). Furthermore, many recent studies have shown that species discovery is still ongoing within this group (Minter et al. 2017), and that species thought to be widespread are often species-complexes composed of taxa with much narrower geographic ranges (Nielsen et al. 2018). Future, fine-scale fieldwork efforts targeting the many undersampled regions across the subcontinent where the *B. mossambicus* species group is likely to occur, combined with population genetic/phylogenomic methods, will be necessary to better investigate the presence of hybridization within *Breviceps*. We are optimistic that future studies scrutinizing morphological data (both morphometric and anatomical, i.e., via CT-scanning) of large numbers of genotyped *B. mossambicus* group samples will reveal diagnostic morphological differences between species and/or populations that are otherwise difficult to discern by individual specimens (Fig. 2).

As mentioned above, there is considerable genetic structure within *B. ombelanonga*, as well as among the four most closely related members of the *B. mossambicus* group (Fig. 1B, C). The Great Escarpment is a major topographical feature of southern Africa that separates the central plateau from coastal plains semi-continuously from Angola in the northwest, south through Namibia and South Africa, before petering out along the border of Zimbabwe and Mozambique in the northeast. This feature is coincident with changes in habitat and climate as one moves from the coast inland, and is consequently reflected in the distribution and diversification of various organisms (Clark et al. 2011; Nielsen et al. 2018). The western and central populations of *B. ombelanonga*, for example, are separated by the escarpment, although further study is needed to verify that the genetic structure we observed (between all three populations) is not just an effect of isolation by distance, compounded by limited sampling. Unfortunately, this is not unique to the *B. mossambicus* group. Many recent studies on other herpetofauna have stated that large sampling gaps across sub-Saharan Africa may cause misleading biogeographic conclusions (Medina et al. 2016; Jongsma et al. 2018). The central and eastern localities of *B. ombelanonga*, as well as the latter from either *B. adspersus* or *B. poweri*, may be separated by drainage basins; however, with no contemporary sampling across regions spanning hundreds of kilometers, it is difficult to test these broad biogeographic hypotheses. Many recent initiatives have improved the current state of knowledge of Angola’s herpetofauna, as well as to identify priority areas for future field survey work (Ceríaco et al. 2014, 2016, 2018; Conradie et al. 2016; Heinicke et al. 2017; Marques et al. 2018; Baptista et al. 2019; Butler et al. 2019; Ernst et al. 2020), yet these efforts have still only scratched the surface. Additional, comprehensive field surveys, particularly those with focused/specialized efforts to record hard-to-find, seasonal, and/or fossorial taxa (e.g., by deploying pitfall traps, drift fence arrays, artificial refuges, etc., for an extended period of time or repeatedly throughout the year), should be priorities in the near future.

## Supplementary Material

XML Treatment for Breviceps ombelanonga

## Footnotes

We here provide only the usages that have been applied to Angola populations, albeit with some inherent uncertainty given the pervasive morphological similarity among members of the B. mossambicus group.

## Appendix 1

**Additional *Breviceps* material examined**

***Breviceps* sp.**: **Angola**: Lunda Sul Province: Alto Chicapa (MD 5426, 5865); Rives du lac Calundo (MD 5599); Moxico Province: Cazombo (MD 5770; MCZ A-35892–893); Luso: Calombe (IICT 339–1959, 404–1959, 453-6–1959); Benguela Province: Benguela (BMNH 1906.10.8.10-11); Ebanga (MHNC 90.008, 90.009); Chimbassi [= Chimbasse] (ZSM 173/1953); Quissange (BMNH 1887.3.23.5); Huambo Province: Bimbi (MCZ A-23721).

***B. adspersus***: **Botswana**: Serowe (PEM A4800); **Namibia**: Damaraland (ZMB 6294 [lectotype]), Okahandja (PEM A4723); **South Africa**: Limpopo Province: Waterpoort (PEM A14226); Mpumalanga Province: Botshabelo (ZMB 10087 [paralectotype]); Northern Cape Province: Rooipoort (PEM A8001–2, PEM A9431, PEM A9433–4), Tswalu (PEM A9444), Kuruman River Reserve (PEM A13883).

***B. mossambicus***: **Malawi**: Mount Mulanje (PEM A7861); **Mozambique**: Cabo Delgado Province: Balama (PEM A11021); Nampula Province: Insula Mossambique (ZMB 75399–400 [syntypes]), Mount Namuli (PEM A11310), Mount Ribaue (PEM A11362), Ribaue town (PEM A13952, PEM A13956), Nagonha Village (PEM A6717); Niassa Province: Lichinga (PEM A14008); Zambezi Province: Mount Lico (PEM A13725–6); **Tanzania** (ZMB 24793).

***B. poweri***: **Democratic Republic of the Congo**: Lualaba Province: Kalakundi (PEM A8453–6); Haut-Katanga Province: Sakania (UF Herp 27586); **Mozambique**: Nampula Province: Ribaue town (PEM A13957); **Zambia**: Northern Province: Mporokoso (PEM A2794); Northwestern Province: Solwezi (CAS 196527); **Zimbabwe**: Melsetter (PEM A4735).

## Supplementary materials

Supplementary material 1 Table S1. Morphological data used to perform PCAs Data type morphological data Explanation note Morphological data used to perform PCAs. See Table 1 and Appendix 1 for sample information. Specimens were derived from localities within the core geographic range of each species, as supported by the phylogenetic results of Nielsen et al. 2018. All were examined to confirm the presence of traits diagnostic for *B. adspersus*, *B. mossambicus*, or *B. poweri*, respectively. https://binary.pensoft.net/file/467836This dataset is made available under the Open Database License (http://opendatacommons.org/licenses/odbl/1.0/). The Open Database License (ODbL) is a license agreement intended to allow users to freely share, modify, and use this Dataset while maintaining this same freedom for others, provided that the original source and author(s) are credited.Stuart V. Nielsen, Werner Conradie, Luis M. P. Ceríaco, Aaron M. Bauer, Matthew P. Heinicke, Edward L. Stanley, David C. Blackburn
